# Correction to ‘Ku–DNA binding inhibitors modulate the DNA damage response in response to DNA double-strand breaks’

**DOI:** 10.1093/narcan/zcae036

**Published:** 2024-08-01

This is a correction to: Pamela L Mendoza-Munoz, Navnath S Gavande, Pamela S VanderVere-Carozza, Katherine S Pawelczak, Joseph R Dynlacht, Joy E Garrett, John J Turchi, Ku–DNA binding inhibitors modulate the DNA damage response in response to DNA double-strand breaks, *NAR Cancer*, Volume 5, Issue 1, March 2023, zcad003, https://doi.org/10.1093/narcan/zcad003

In Figure 5B of the original published version, the immunofluorescent images of the vehicle controls at 0.25h, 0.5h, 1h, and 6h hours were accidentally duplicated. The mistake occurred during figure assembly when placeholder images were not replaced with the actual images from the corresponding time points.

To correct the scientific record, the authors have provided the original images in the Supplementary data and a revised Figure 5 below.

This correction does not affect the results, discussion and conclusions presented in the article.

These details have been corrected only in this correction notice to preserve the published version of record.

## Supplementary data

Supplementary Data are available at NAR Cancer Online.


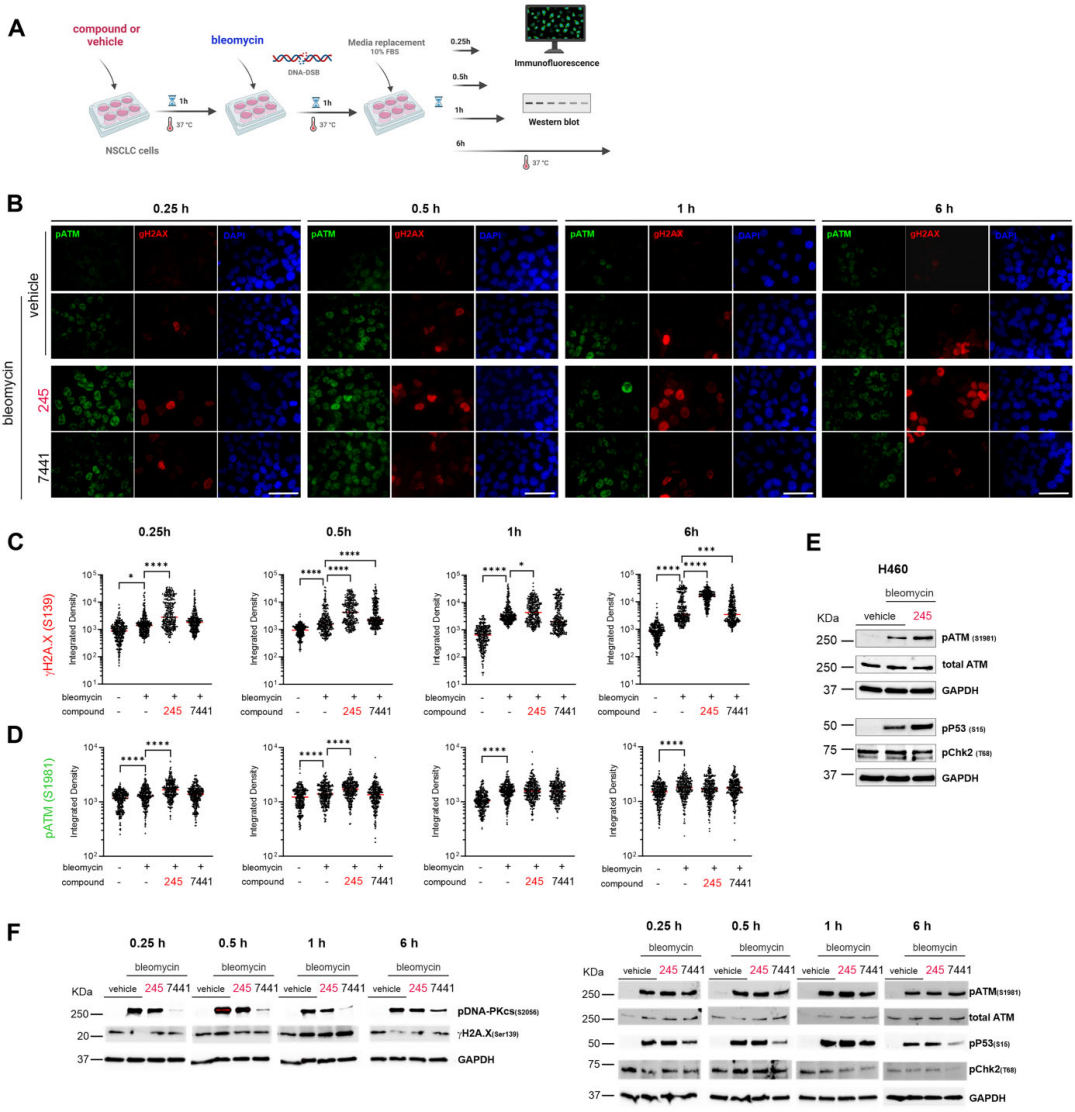


## Supplementary Material

zcae036_Supplemental_Files

