# Supplementary figures and images for: Correction to ‘Ku–DNA binding inhibitors modulate the DNA damage response in response to DNA double-strand breaks’
Source: NAR Cancer. 2024 Aug 1;6(3):zcae036. doi: 10.1093/narcan/zcae036 (PMC11292405; doi:10.1093/narcan/zcae036)

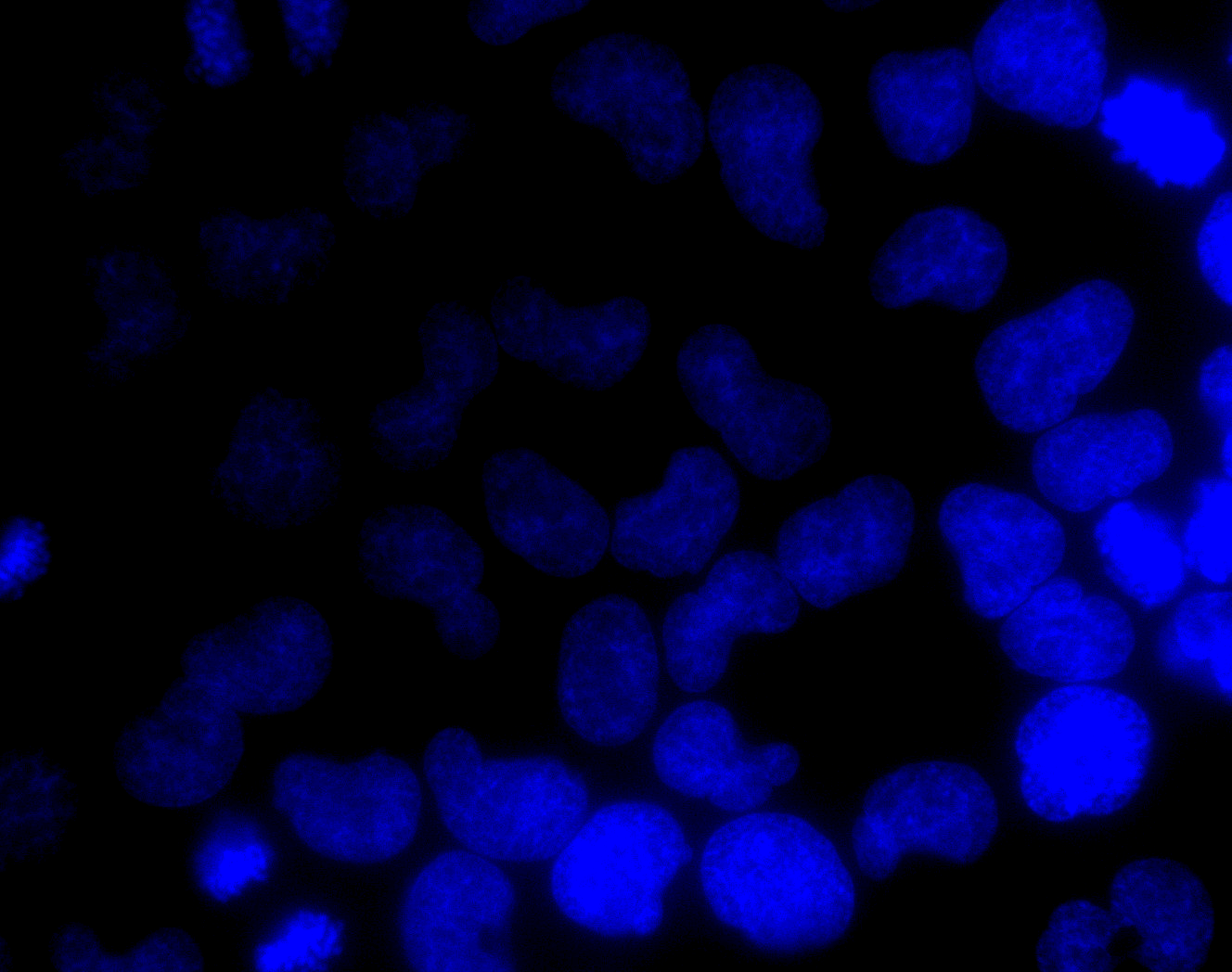

Supplement: zcae036_Supplemental_Files [file zcae036_supplemental_files.zip › 001- 0.25h/60x- Untreated- 0.25h AT_001_g_dapi.tif]

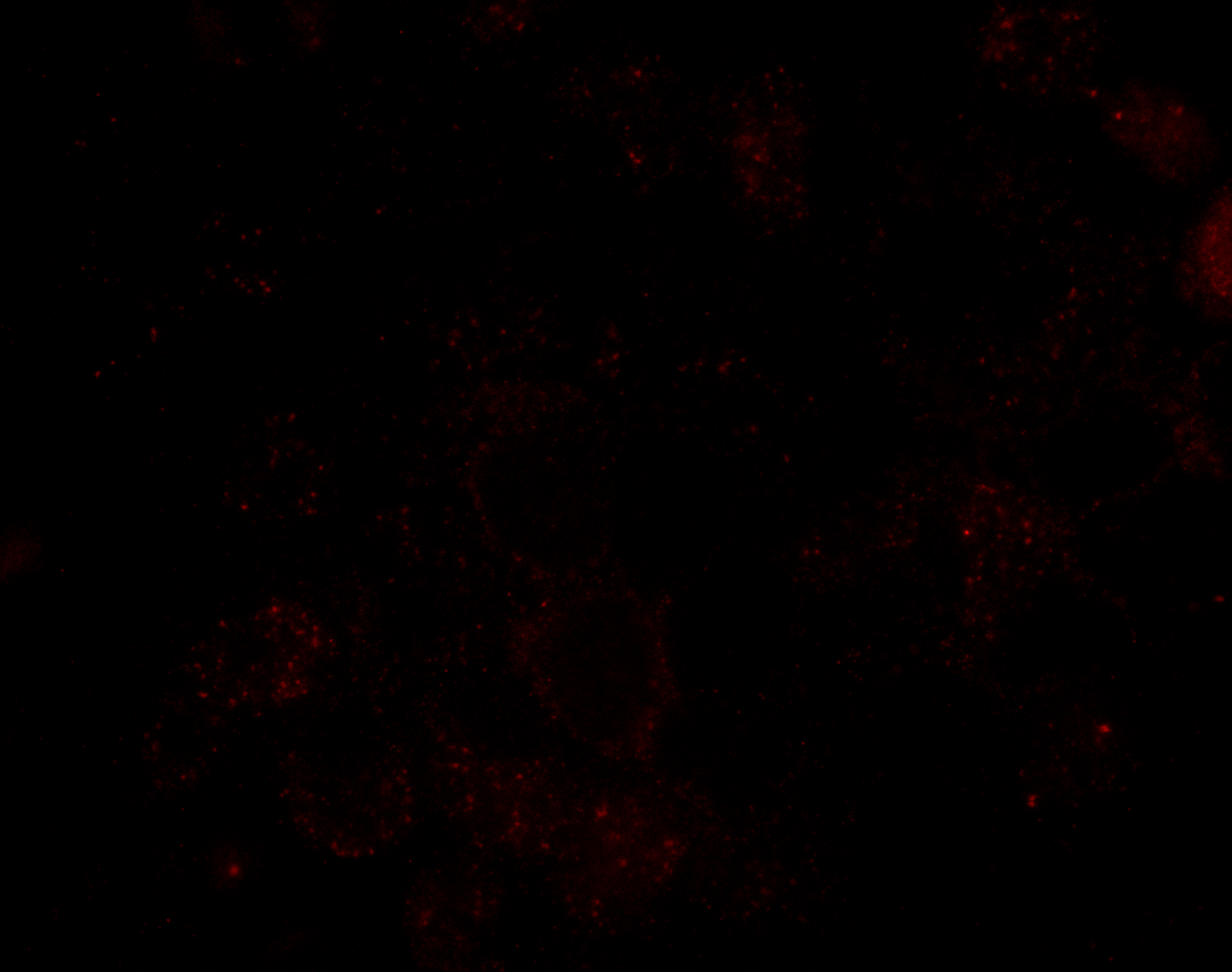

Supplement: zcae036_Supplemental_Files [file zcae036_supplemental_files.zip › 001- 0.25h/60x- Untreated- 0.25h AT_001_g_gH2A.X.tif]

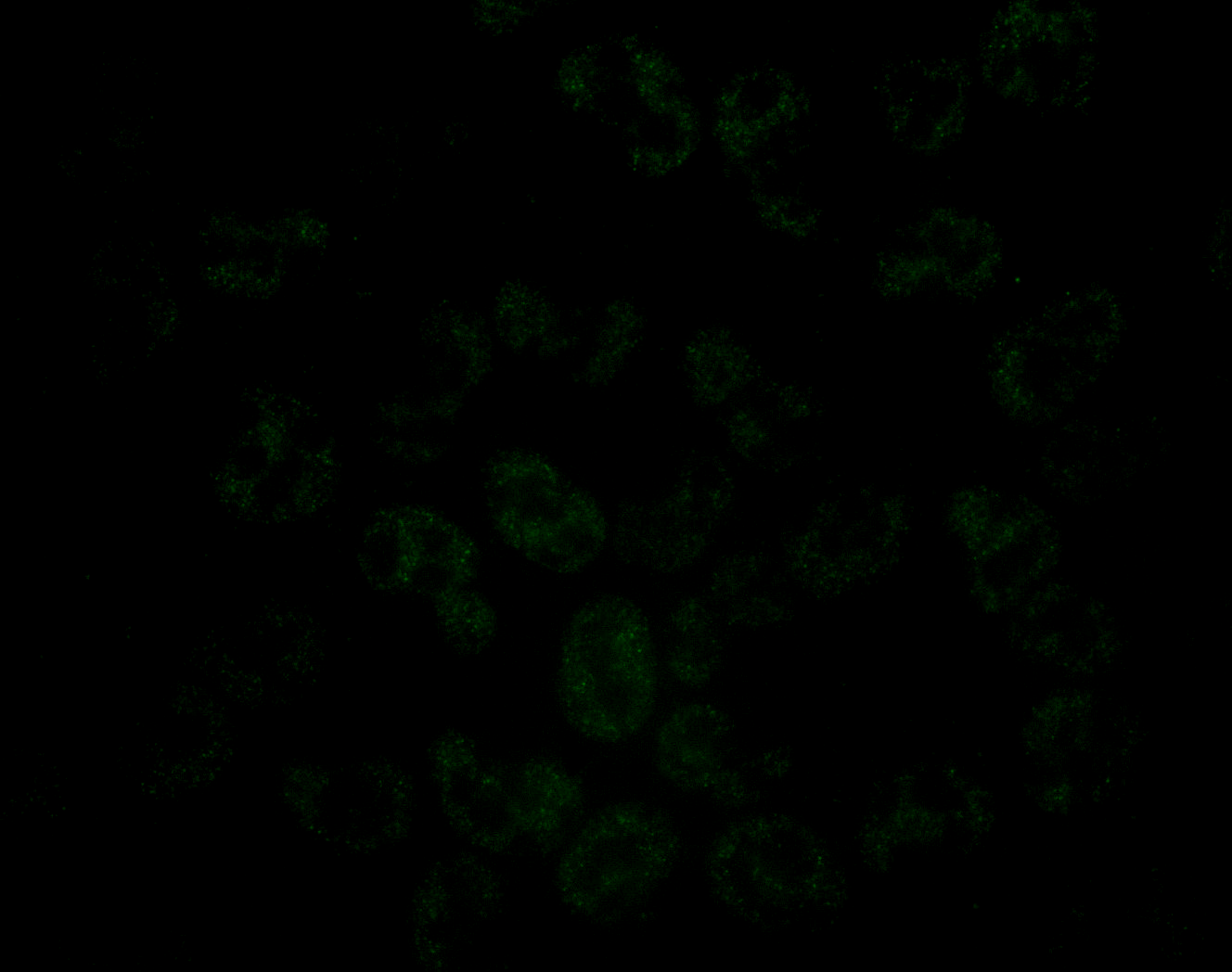

Supplement: zcae036_Supplemental_Files [file zcae036_supplemental_files.zip › 001- 0.25h/60x- Untreated- 0.25h AT_001_g_pATM.tif]

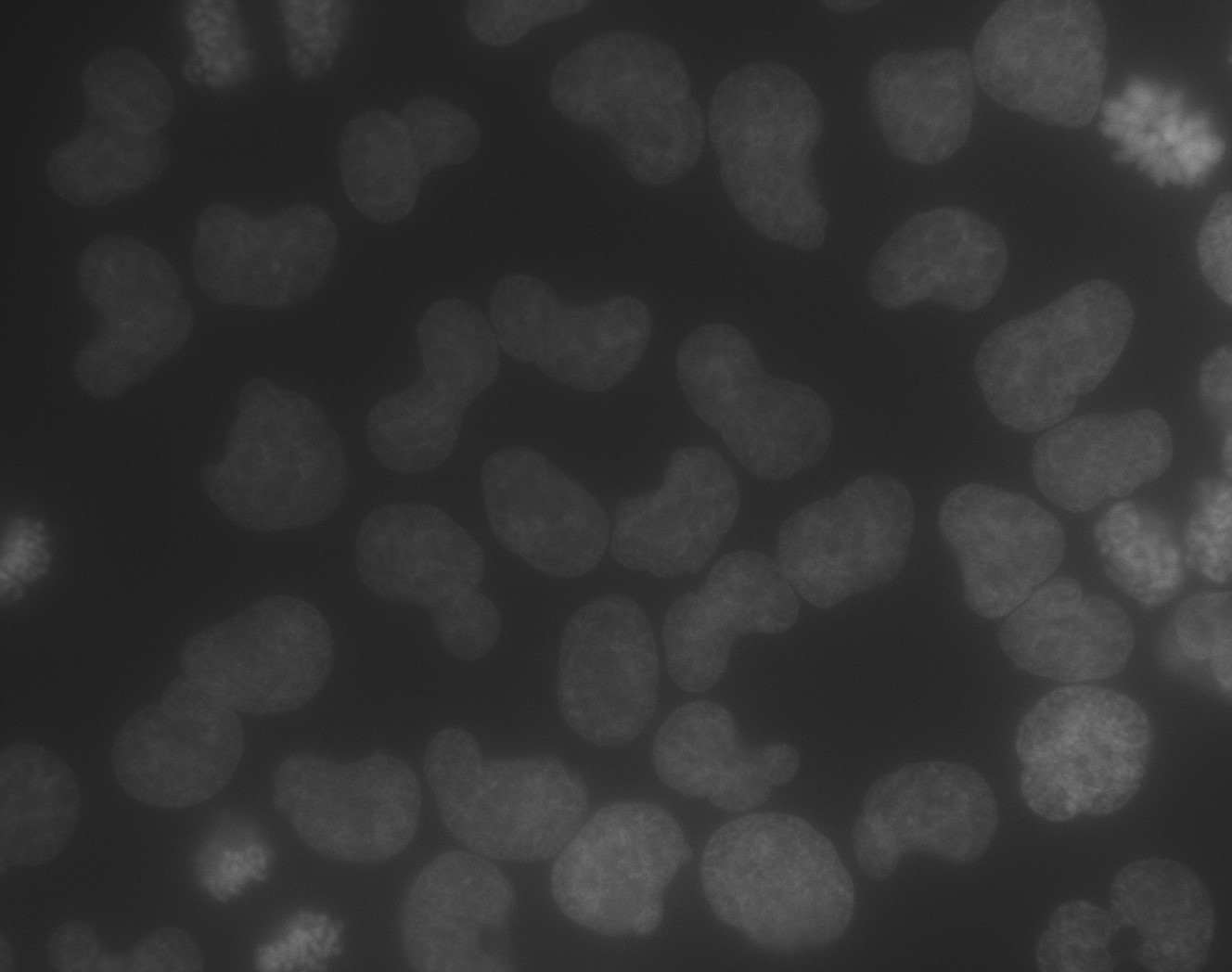

Supplement: zcae036_Supplemental_Files [file zcae036_supplemental_files.zip › 001- 0.25h/60x- Untreated- 0.25h AT_001_g_Top Slide_D_p01_0_A01f16d0.JPG]

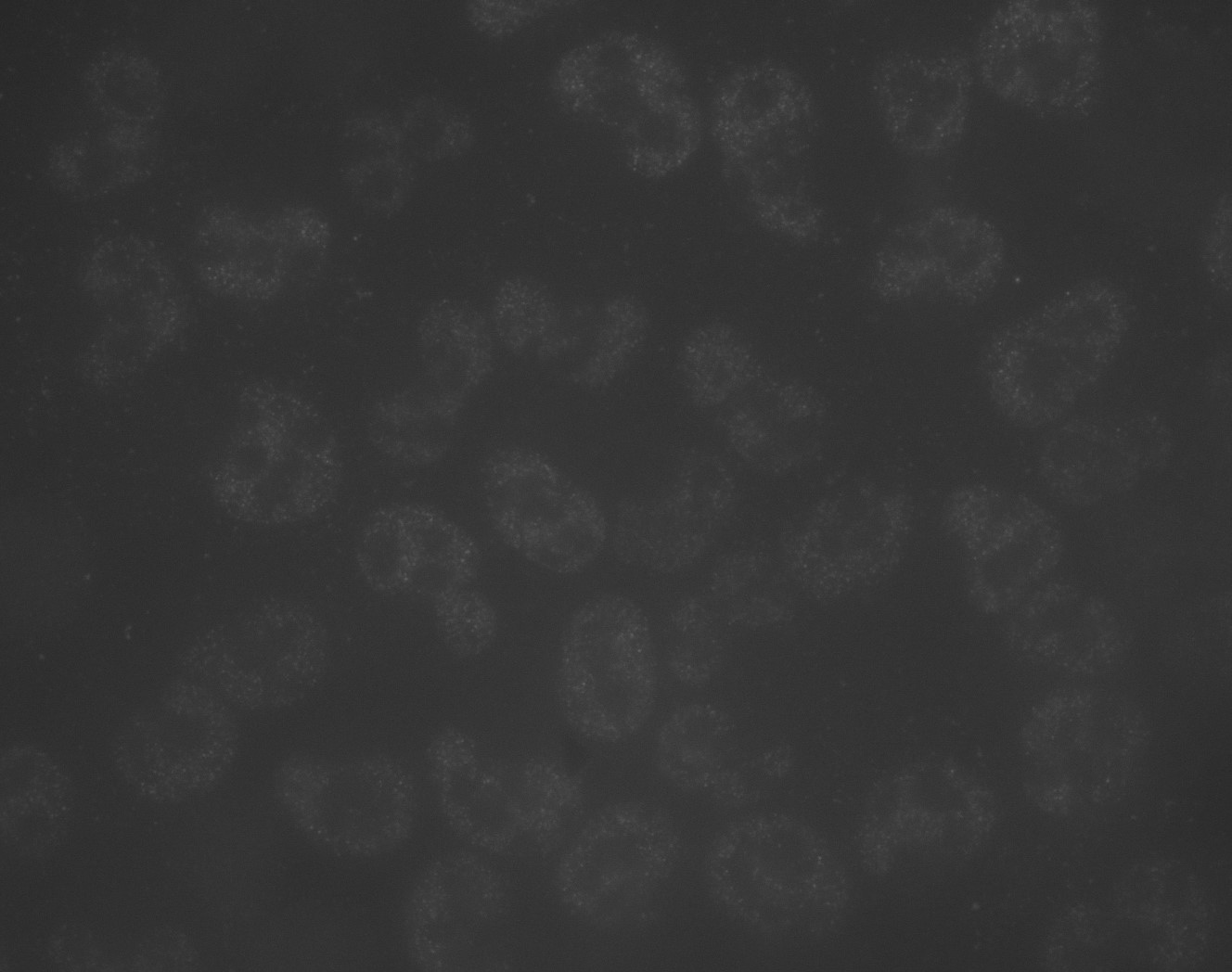

Supplement: zcae036_Supplemental_Files [file zcae036_supplemental_files.zip › 001- 0.25h/60x- Untreated- 0.25h AT_001_g_Top Slide_D_p01_0_A01f16d1.JPG]

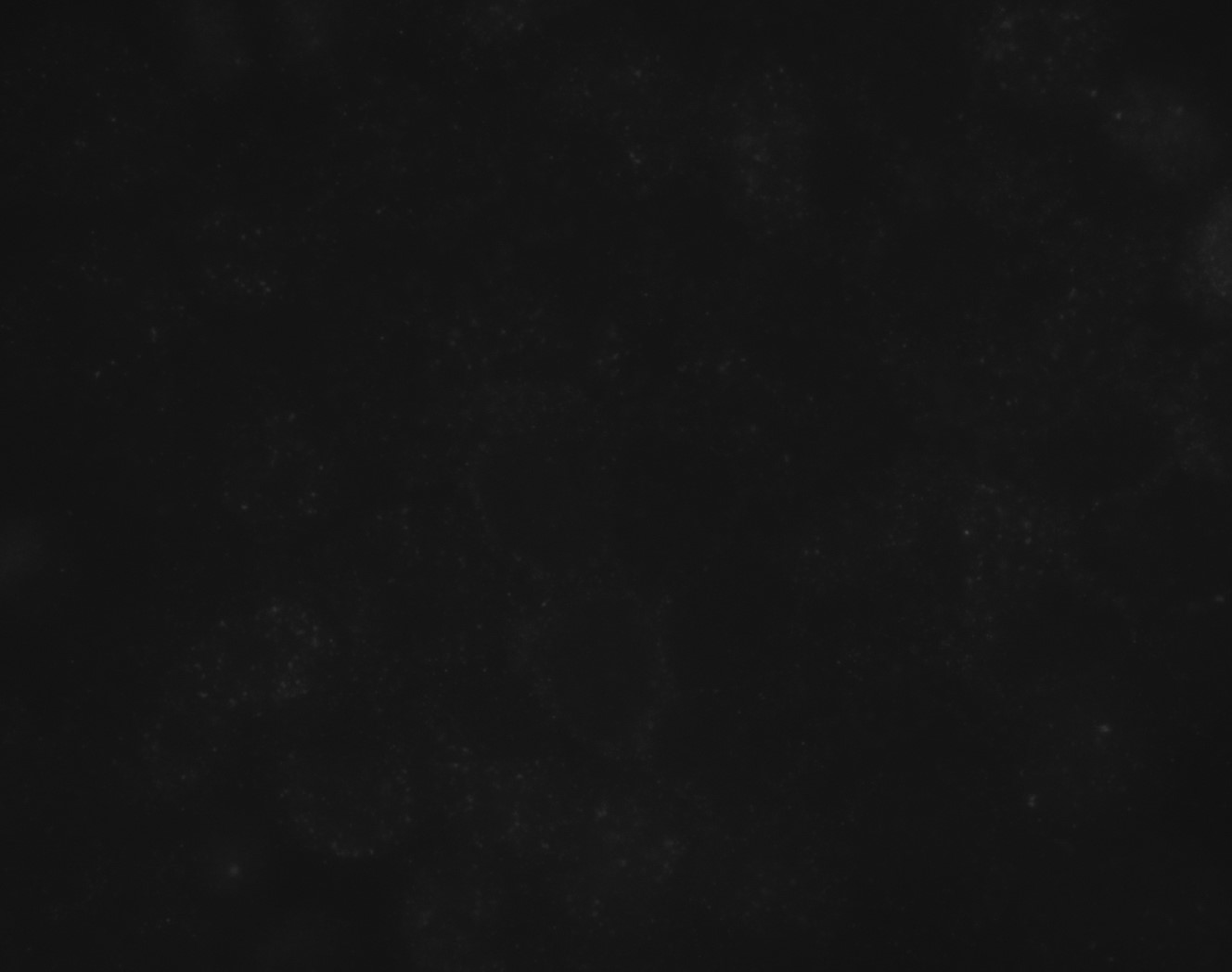

Supplement: zcae036_Supplemental_Files [file zcae036_supplemental_files.zip › 001- 0.25h/60x- Untreated- 0.25h AT_001_g_Top Slide_D_p01_0_A01f16d2.JPG]

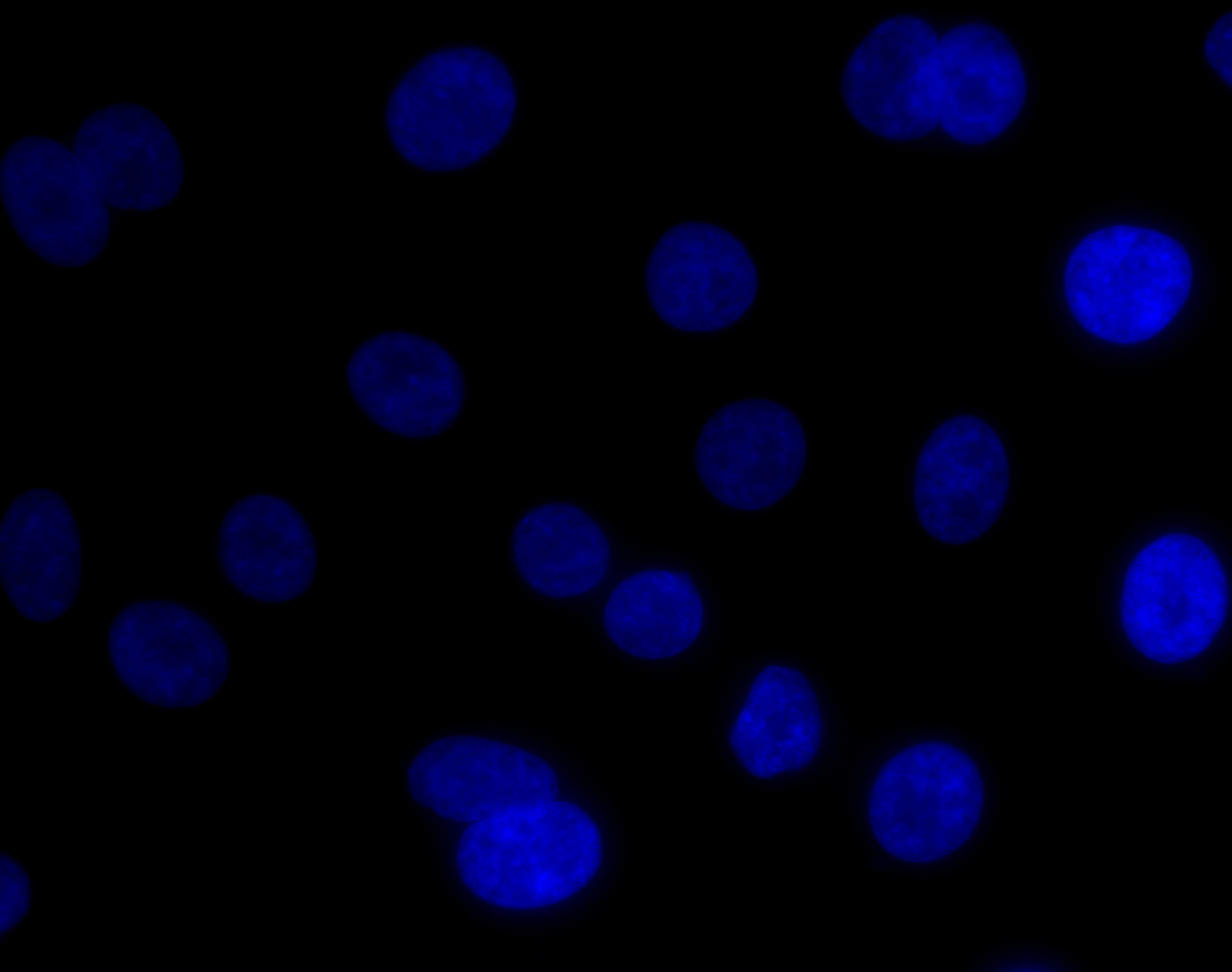

Supplement: zcae036_Supplemental_Files [file zcae036_supplemental_files.zip › 001- 1h/60x- 1h AT- Untreated_g_002_dapi.tif]

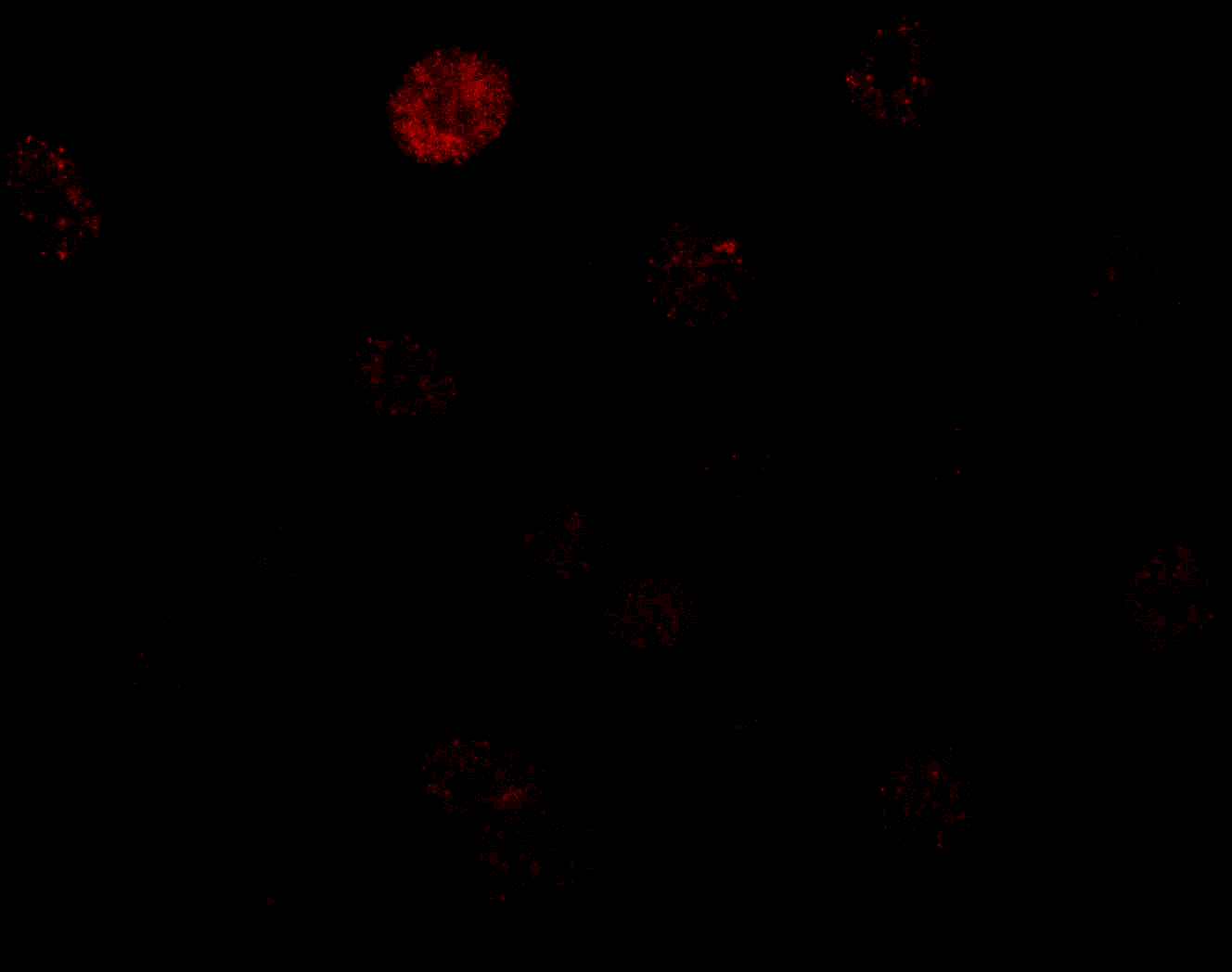

Supplement: zcae036_Supplemental_Files [file zcae036_supplemental_files.zip › 001- 1h/60x- 1h AT- Untreated_g_002_gH2AX.tif]

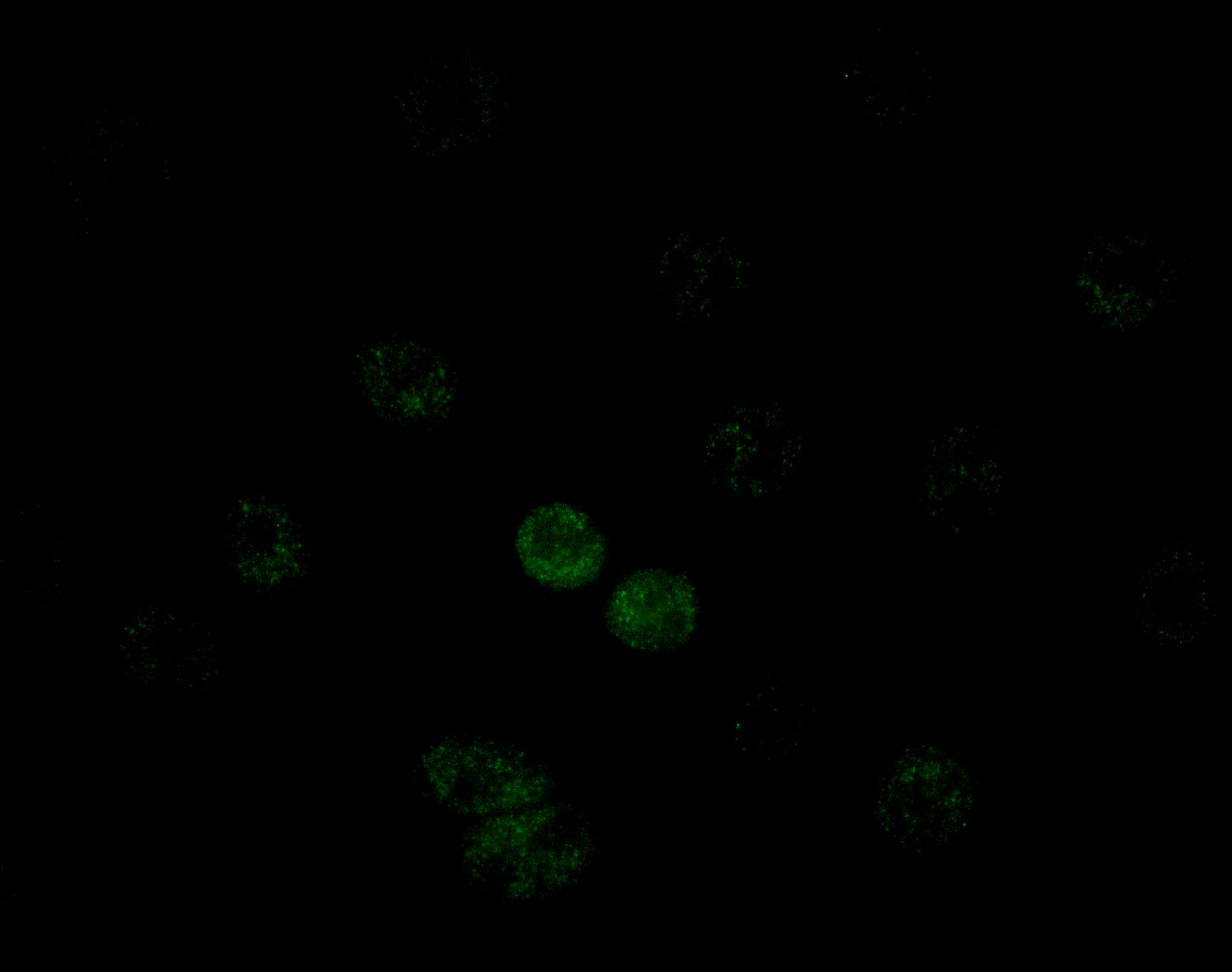

Supplement: zcae036_Supplemental_Files [file zcae036_supplemental_files.zip › 001- 1h/60x- 1h AT- Untreated_g_002_pATM.tif]

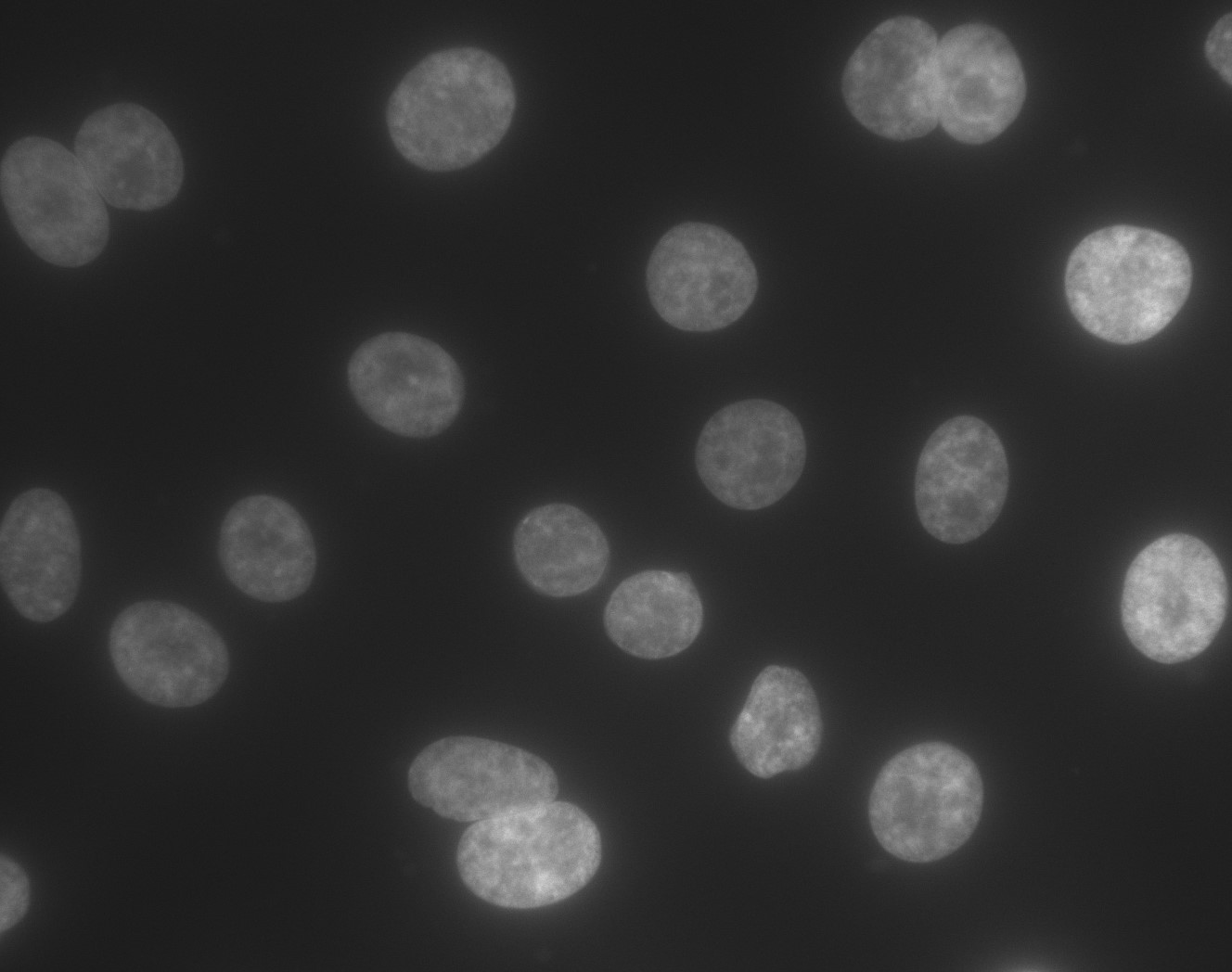

Supplement: zcae036_Supplemental_Files [file zcae036_supplemental_files.zip › 001- 1h/60x- 1h AT- Untreated_g_002_Top Slide_D_p00_0_A01f20d0.JPG]

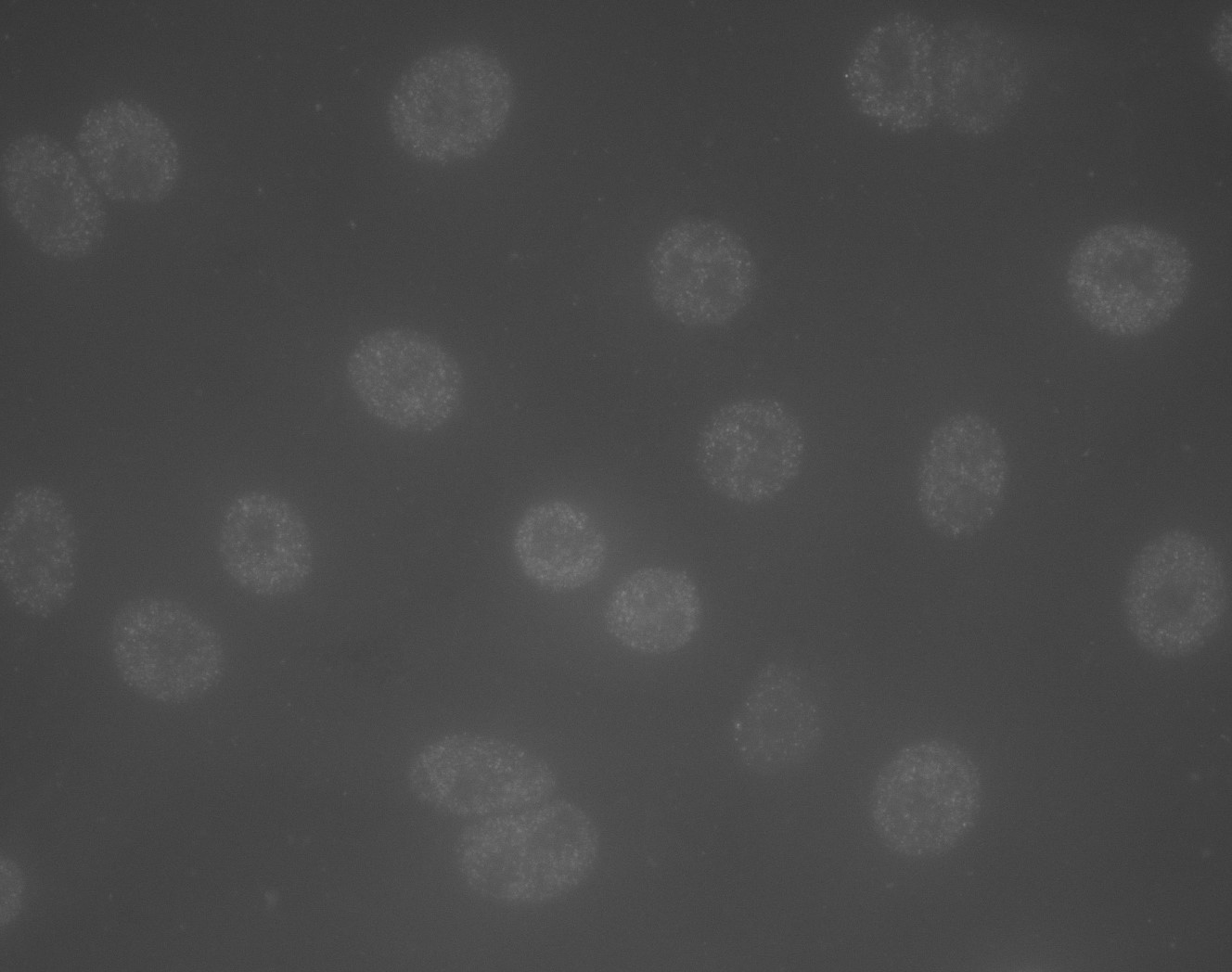

Supplement: zcae036_Supplemental_Files [file zcae036_supplemental_files.zip › 001- 1h/60x- 1h AT- Untreated_g_002_Top Slide_D_p00_0_A01f20d1.JPG]

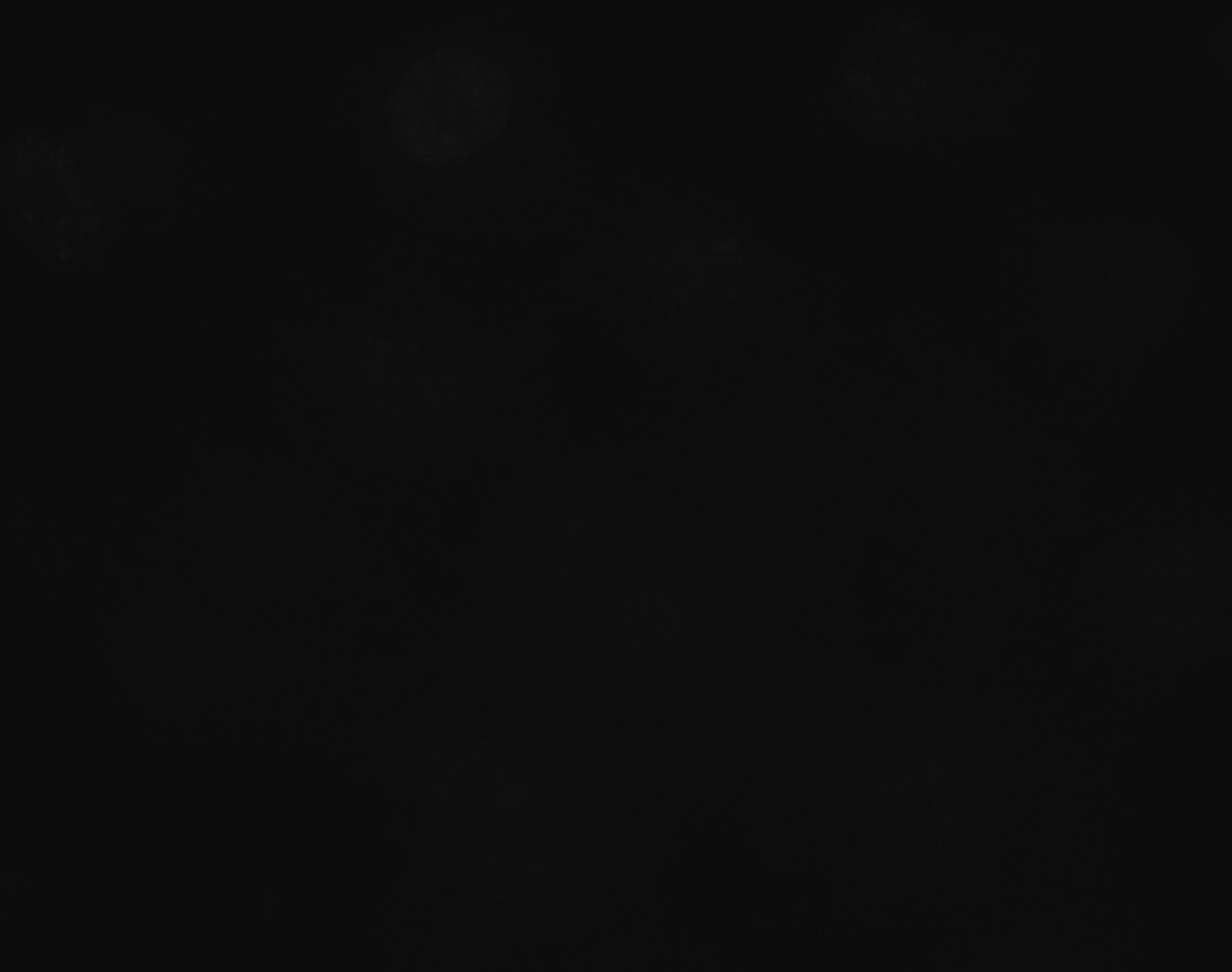

Supplement: zcae036_Supplemental_Files [file zcae036_supplemental_files.zip › 001- 1h/60x- 1h AT- Untreated_g_002_Top Slide_D_p00_0_A01f20d2.JPG]

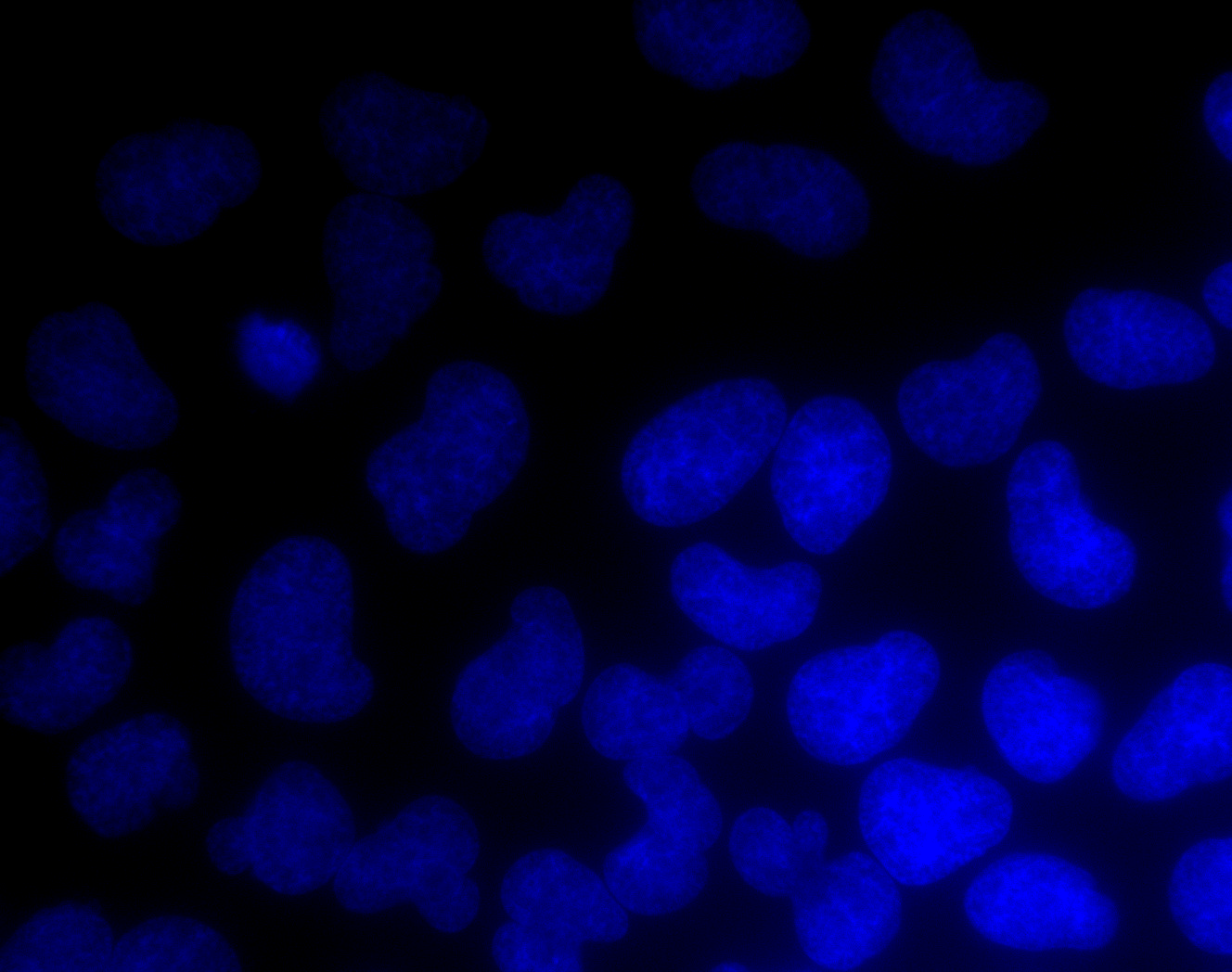

Supplement: zcae036_Supplemental_Files [file zcae036_supplemental_files.zip › 002- 0.5h/60x- Untreated- 0.5h AT_002_g_dapi.tif]

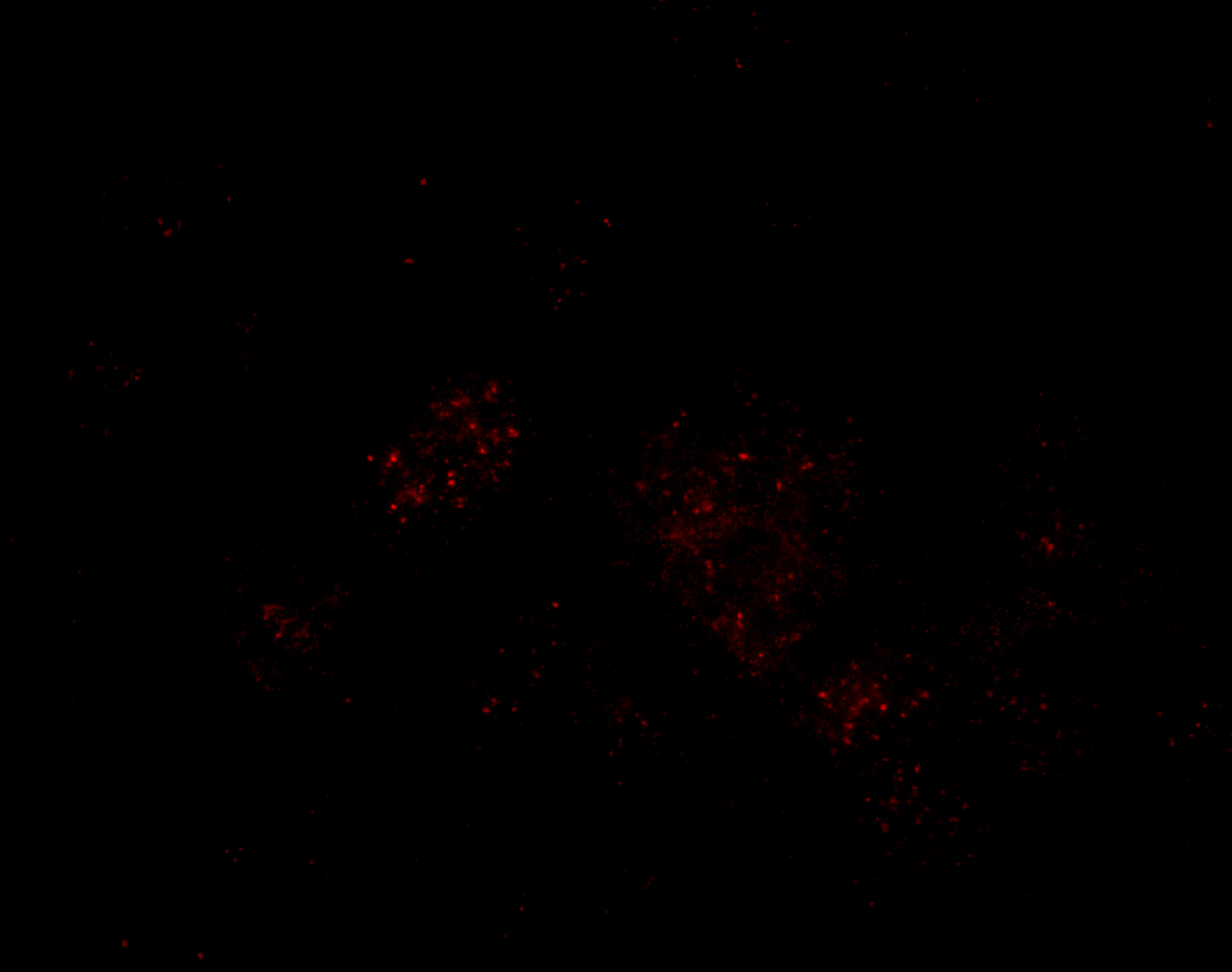

Supplement: zcae036_Supplemental_Files [file zcae036_supplemental_files.zip › 002- 0.5h/60x- Untreated- 0.5h AT_002_g_gH2AX.tif]

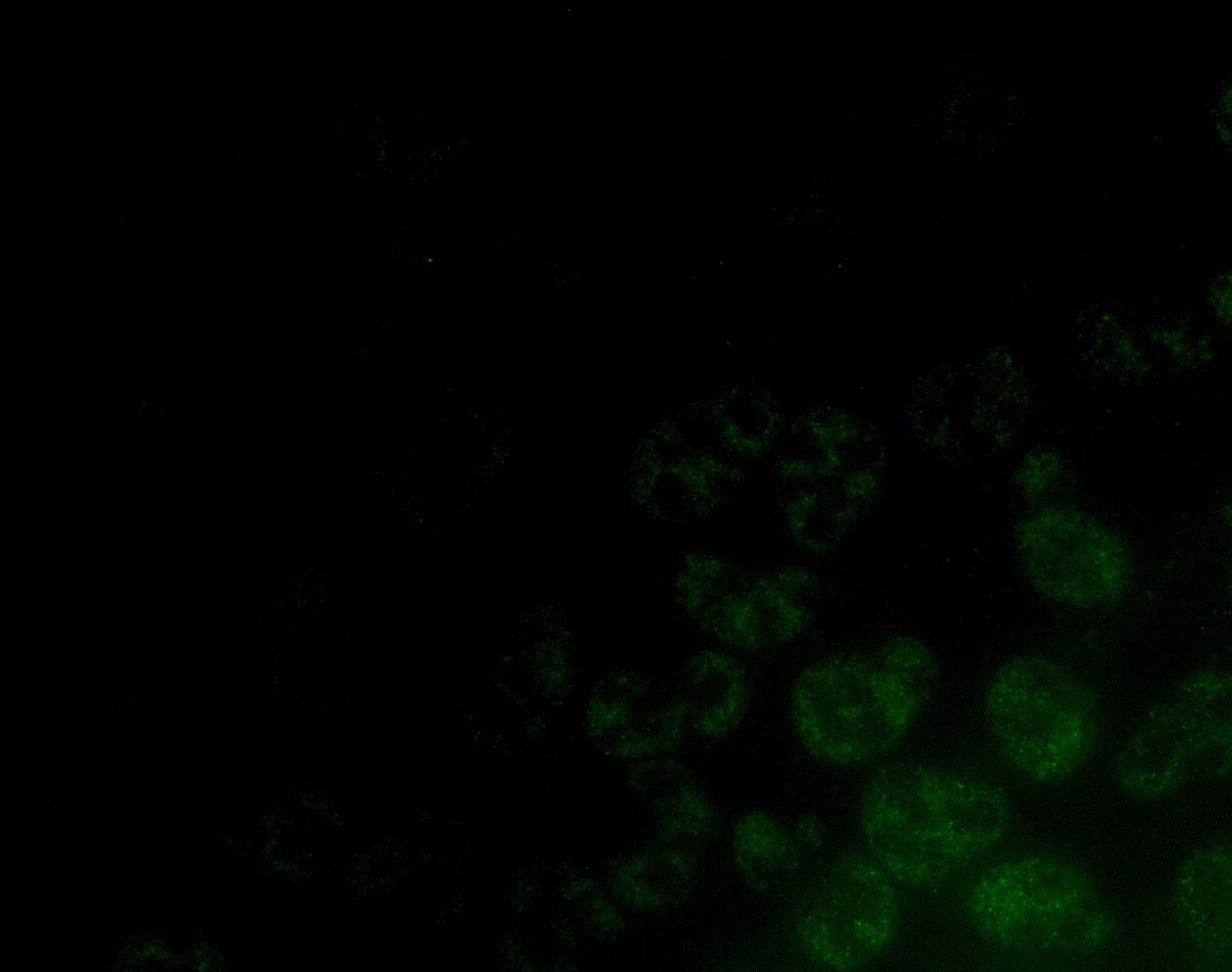

Supplement: zcae036_Supplemental_Files [file zcae036_supplemental_files.zip › 002- 0.5h/60x- Untreated- 0.5h AT_002_g_pATM.tif]

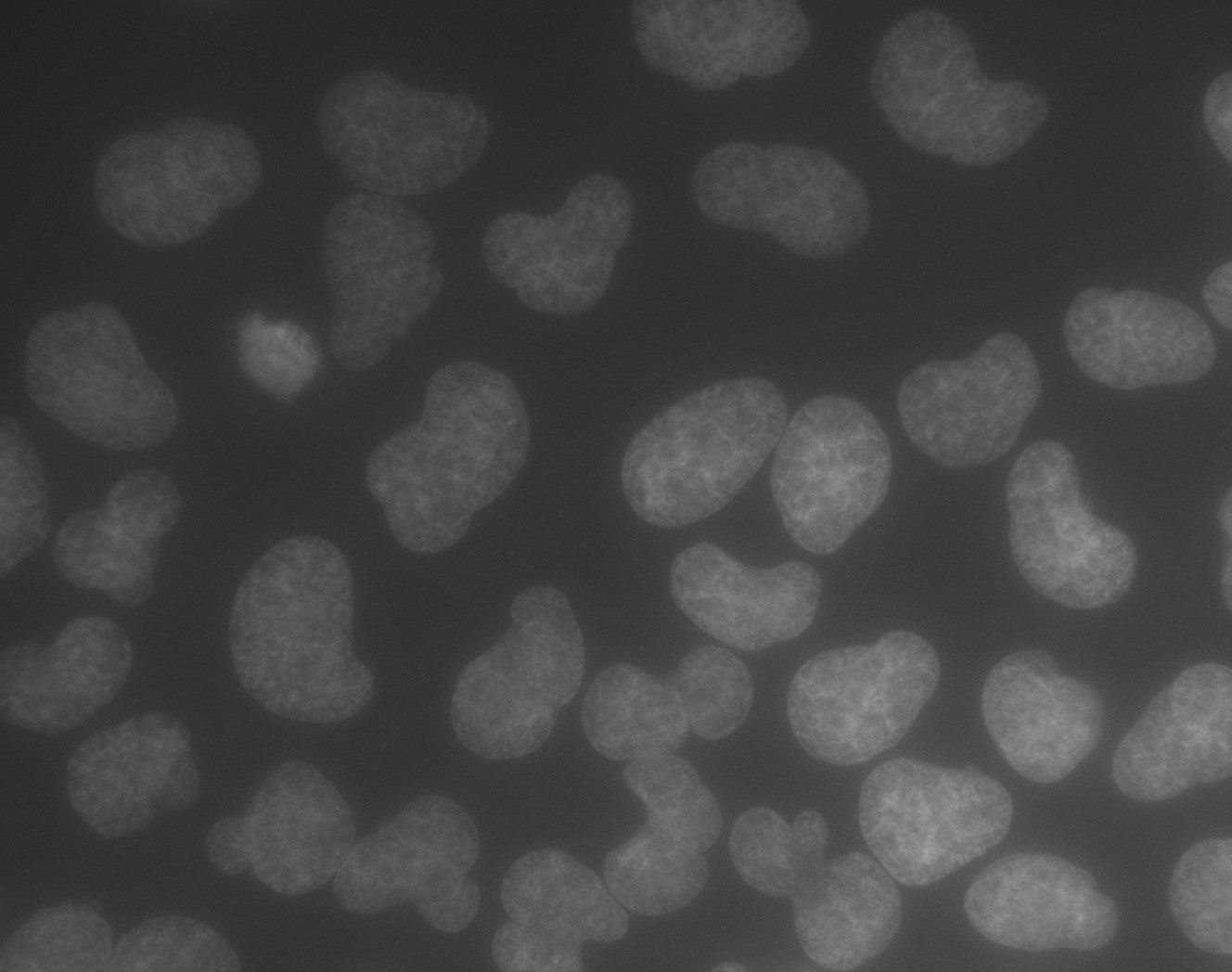

Supplement: zcae036_Supplemental_Files [file zcae036_supplemental_files.zip › 002- 0.5h/60x- Untreated- 0.5h AT_002_g_Top Slide_D_p01_0_A01f17d0.JPG]

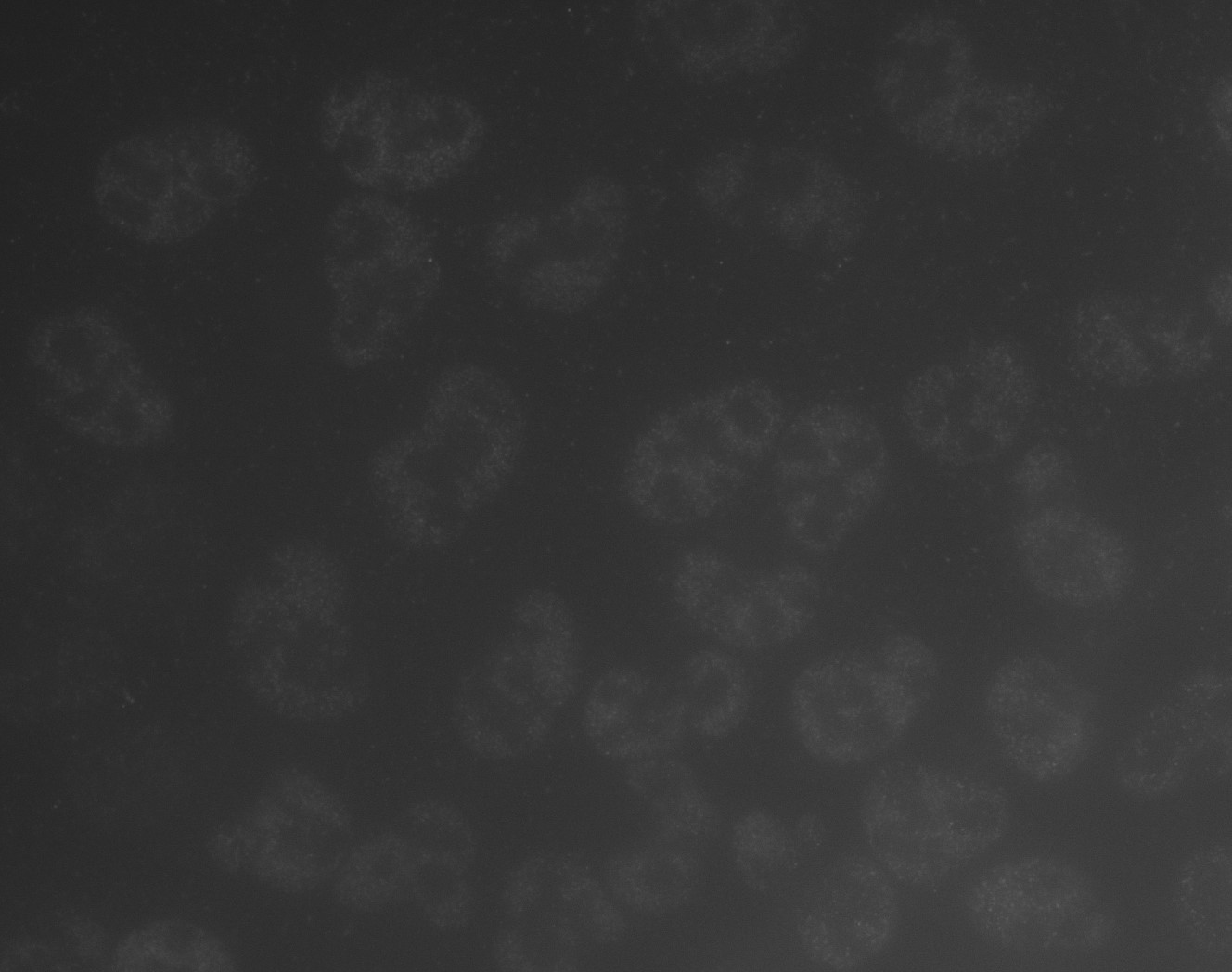

Supplement: zcae036_Supplemental_Files [file zcae036_supplemental_files.zip › 002- 0.5h/60x- Untreated- 0.5h AT_002_g_Top Slide_D_p01_0_A01f17d1.JPG]

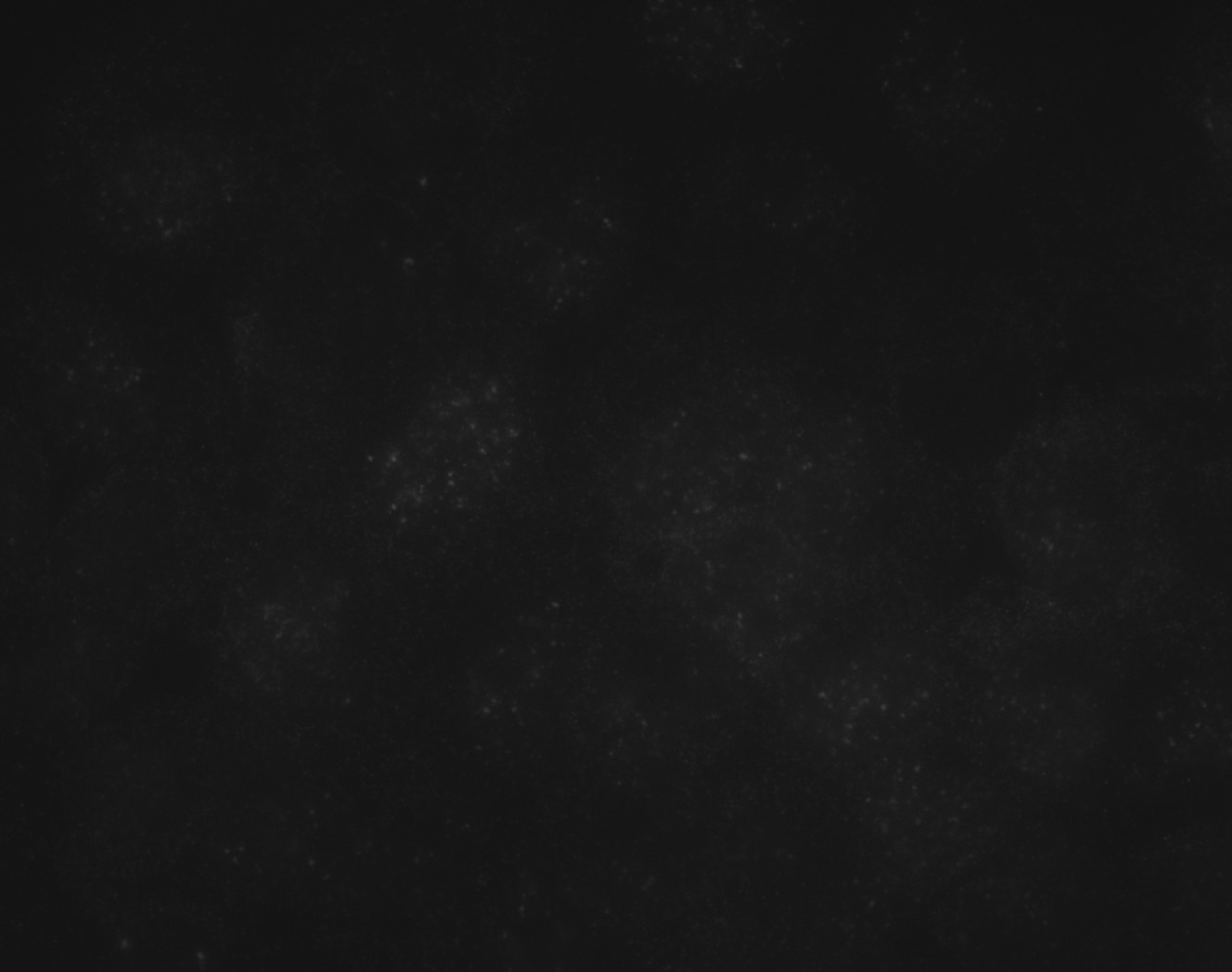

Supplement: zcae036_Supplemental_Files [file zcae036_supplemental_files.zip › 002- 0.5h/60x- Untreated- 0.5h AT_002_g_Top Slide_D_p01_0_A01f17d2.JPG]

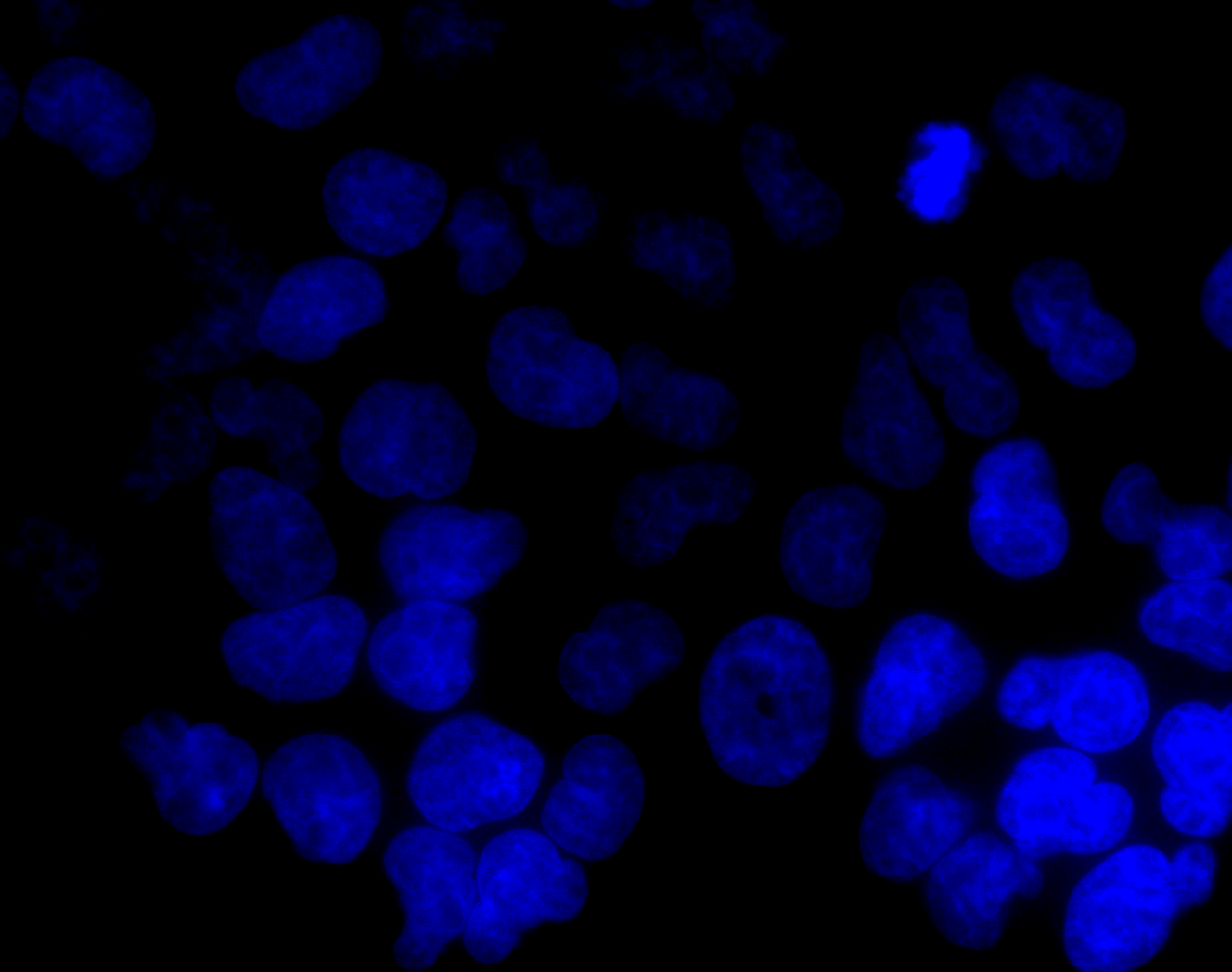

Supplement: zcae036_Supplemental_Files [file zcae036_supplemental_files.zip › 002- 6h/60x- Untreated- 6h AT_002_g_dapi.tif]

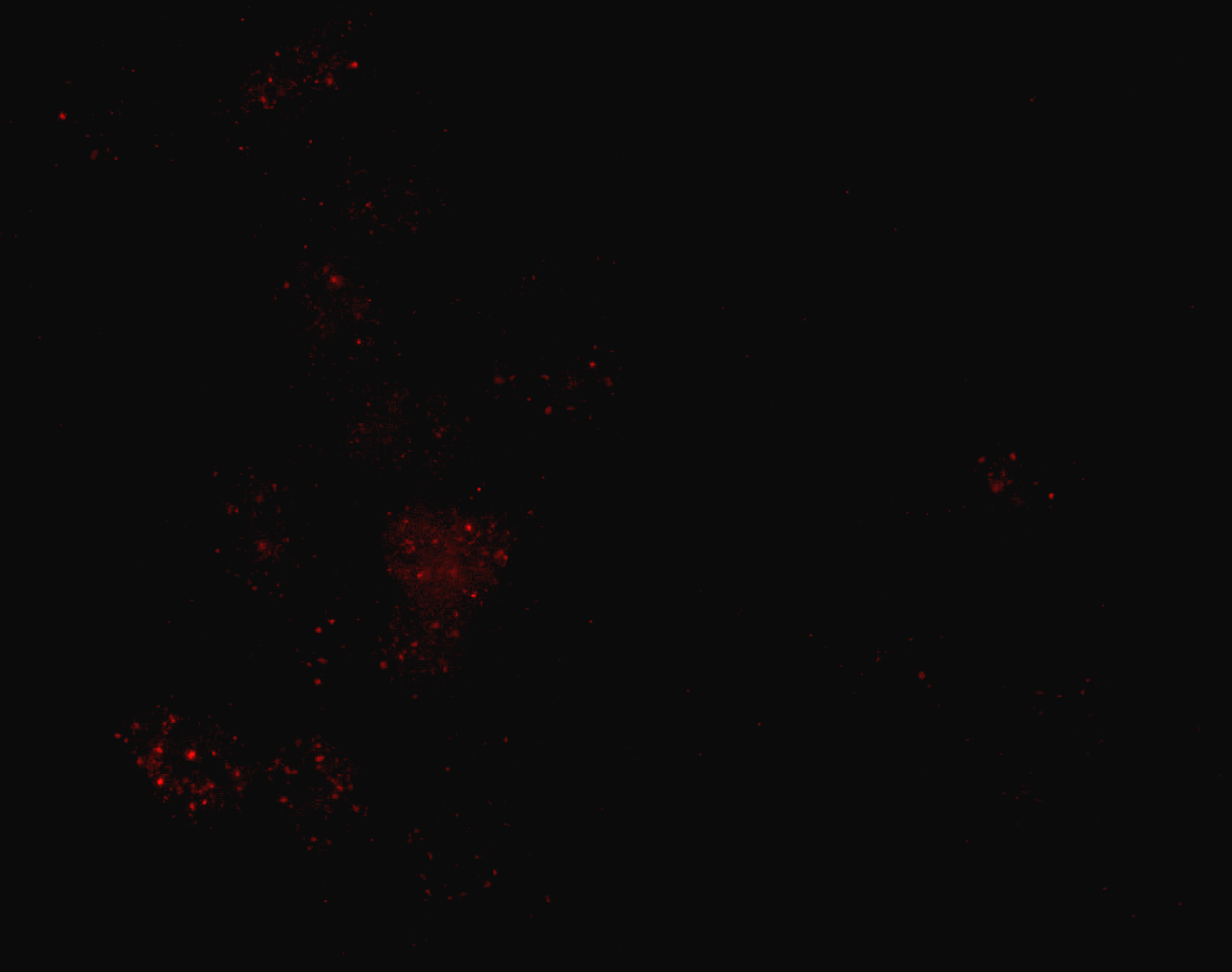

Supplement: zcae036_Supplemental_Files [file zcae036_supplemental_files.zip › 002- 6h/60x- Untreated- 6h AT_002_g_gH2AX.tif]

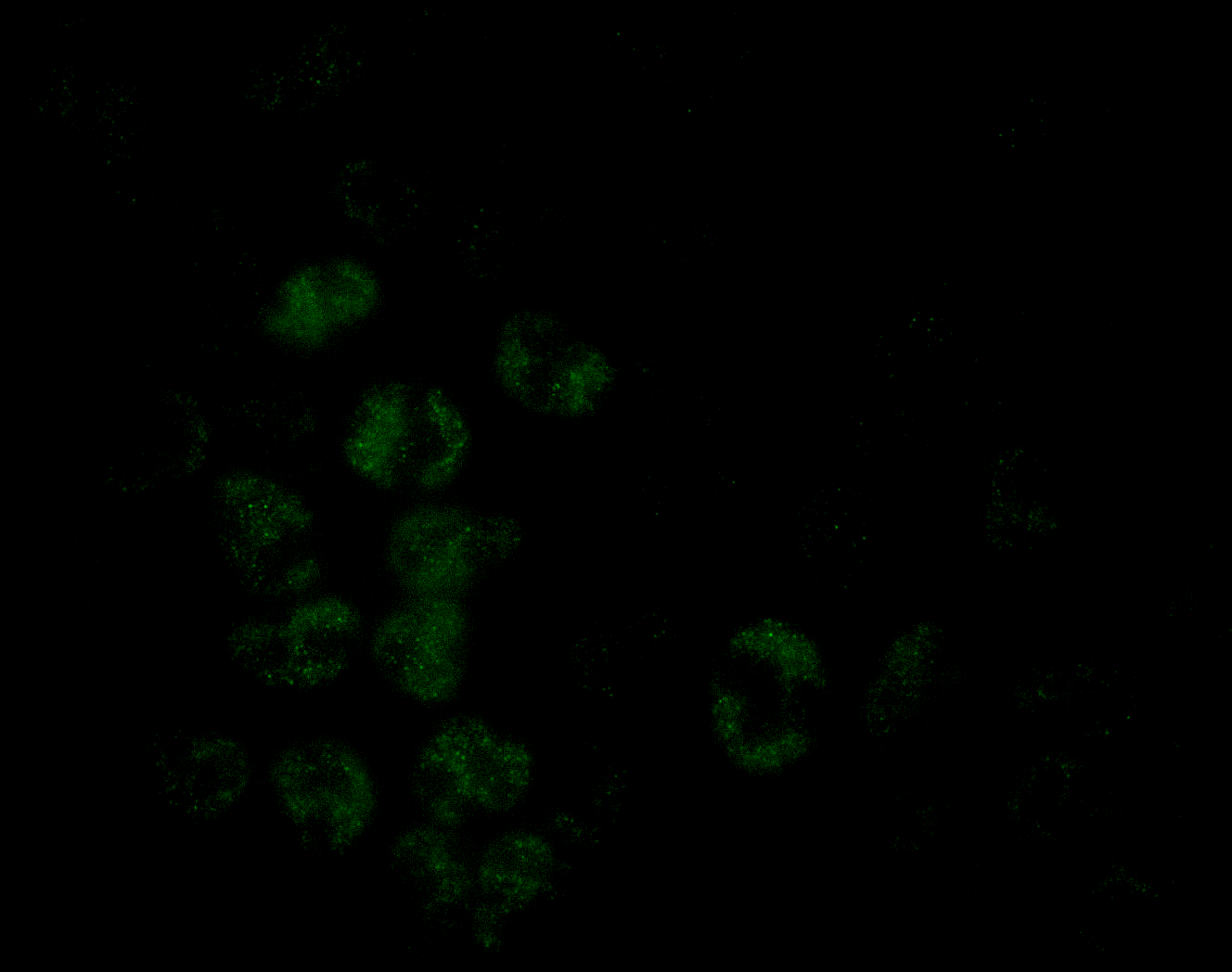

Supplement: zcae036_Supplemental_Files [file zcae036_supplemental_files.zip › 002- 6h/60x- Untreated- 6h AT_002_g_pATM.tif]

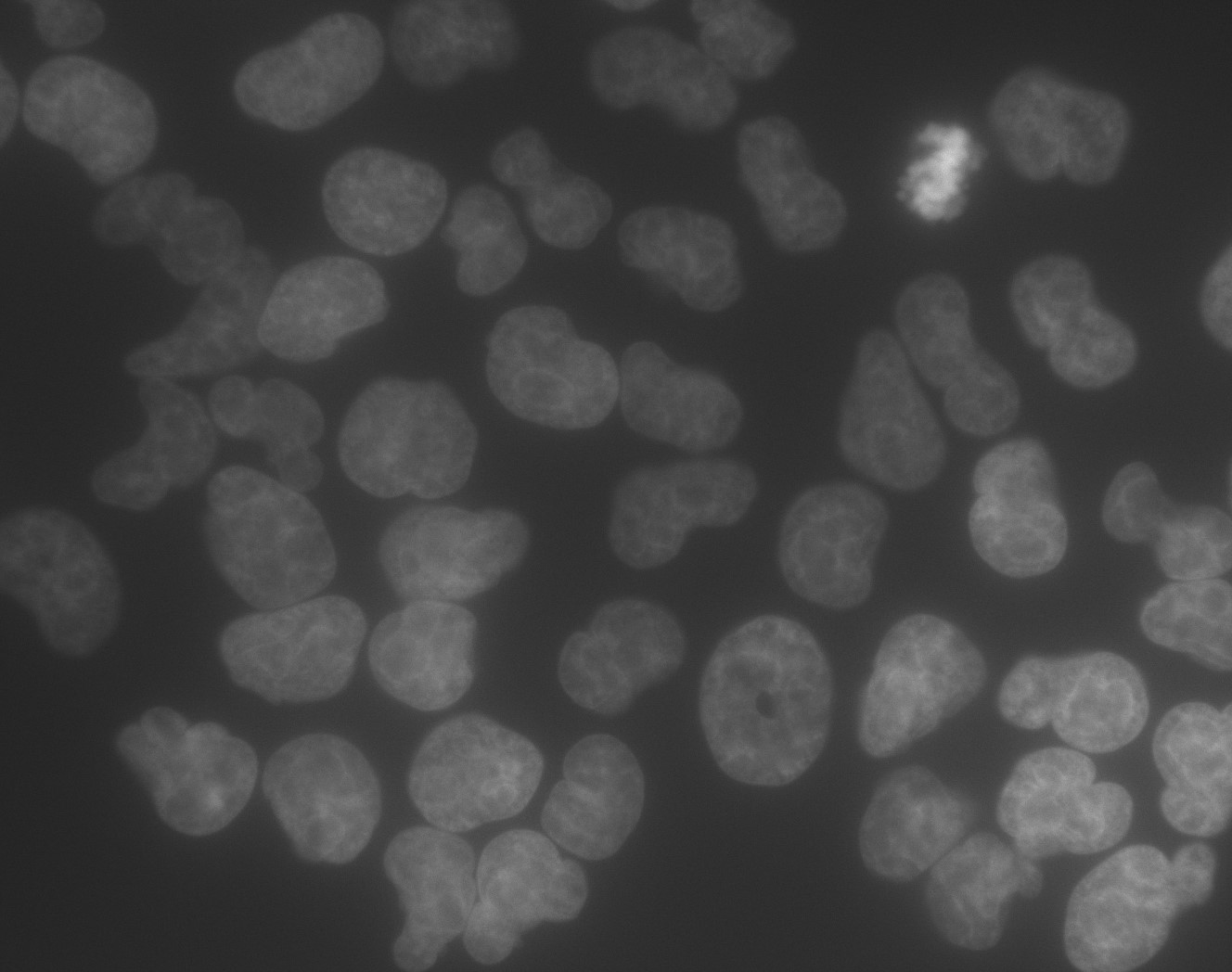

Supplement: zcae036_Supplemental_Files [file zcae036_supplemental_files.zip › 002- 6h/60x- Untreated- 6h AT_002_g_Top Slide_D_p01_0_A01f15d0.JPG]

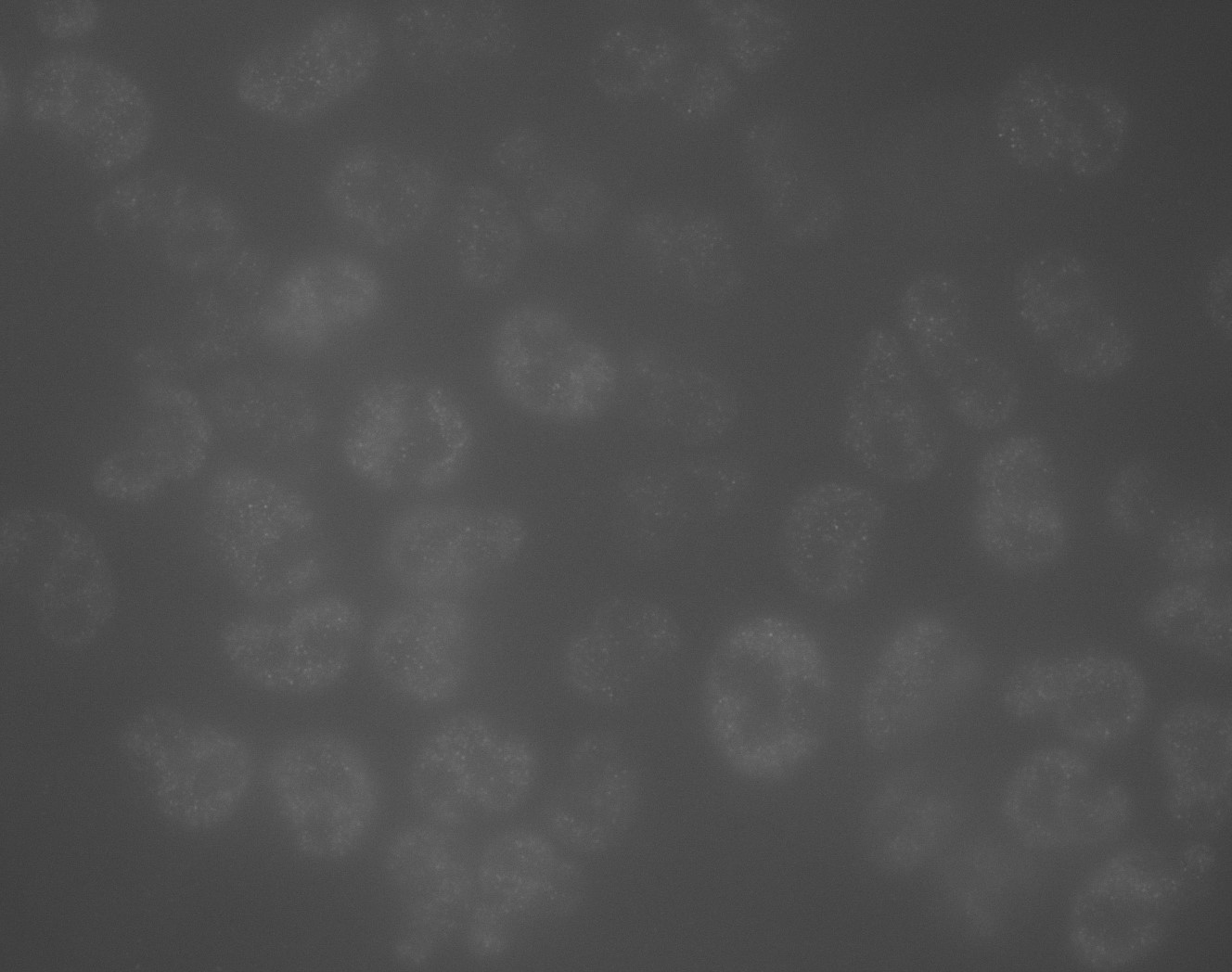

Supplement: zcae036_Supplemental_Files [file zcae036_supplemental_files.zip › 002- 6h/60x- Untreated- 6h AT_002_g_Top Slide_D_p01_0_A01f15d1.JPG]

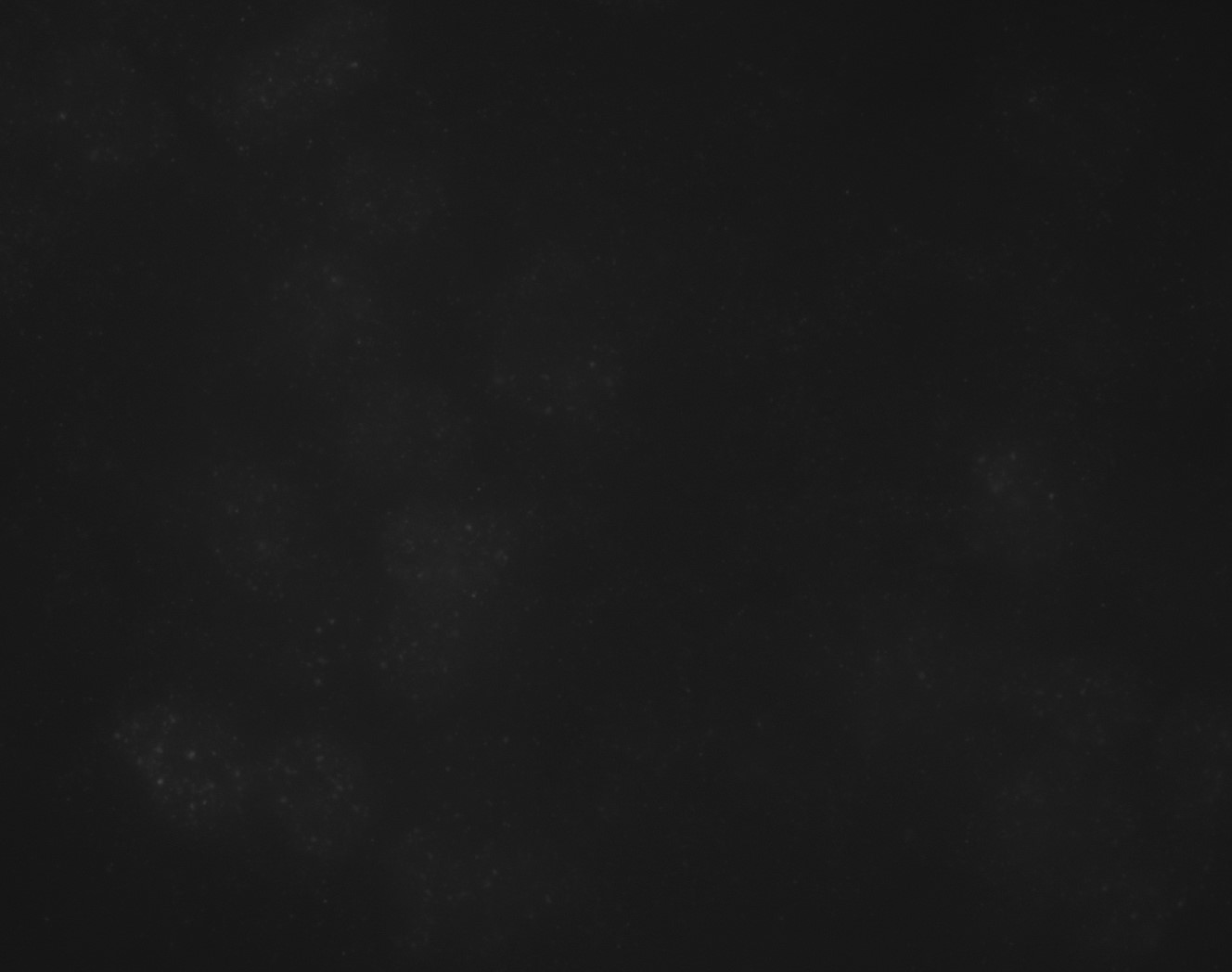

Supplement: zcae036_Supplemental_Files [file zcae036_supplemental_files.zip › 002- 6h/60x- Untreated- 6h AT_002_g_Top Slide_D_p01_0_A01f15d2.JPG]
